# Molecular Malaria Epidemiology: Mapping and Burden Estimates for the Democratic Republic of the Congo, 2007

**DOI:** 10.1371/journal.pone.0016420

**Published:** 2011-01-31

**Authors:** Steve M. Taylor, Jane P. Messina, Carla C. Hand, Jonathan J. Juliano, Jeremie Muwonga, Antoinette K. Tshefu, Benjamin Atua, Michael Emch, Steven R. Meshnick

**Affiliations:** 1 Department of Epidemiology, Gillings School of Global Public Health, University of North Carolina, Chapel Hill, North Carolina, United States of America; 2 Division of Infectious Diseases and International Health, Duke University Medical Center, Durham, North Carolina, United States of America; 3 Department of Geography, University of North Carolina, Chapel Hill, North Carolina, United States of America; 4 Carolina Population Center, University of North Carolina, Chapel Hill, North Carolina, United States of America; 5 Division of Infectious Diseases, University of North Carolina School of Medicine, Chapel Hill, North Carolina, United States of America; 6 Laboratoire National de Reference SIDA et IST (LNRS), Kinshasa, Republique Démocratique du Congo; 7 Ecole de Santé Publique, Faculté de Medecine, Université de Kinshasa, Republique Démocratique du Congo; 8 Programme National de Lutte contre le Paludisme (PNLP), Kinshasa, Republique Démocratique du Congo; Universidade Federal de Minas Gerais, Brazil

## Abstract

**Background:**

Epidemiologic data on malaria are scant in many high-burden countries including the Democratic Republic of the Congo (DRC), which suffers the second-highest global burden of malaria. Malaria control efforts in regions with challenging infrastructure require reproducible and efficient surveillance. We employed new high-throughput molecular testing to characterize the state of malaria control in the DRC and estimate childhood mortality attributable to excess malaria transmission.

**Methods and Findings:**

The Demographic and Health Survey was a cross-sectional, population-based cluster household survey of adults aged 15–59 years in 2007 employing structured questionnaires and dried blood spot collection. Parasitemia was detected by real-time PCR, and survey responses measured adoption of malaria control measures and under-5 health indices. The response rate was 99% at the household level, and 8,886 households were surveyed in 300 clusters; from 8,838 respondents molecular results were available. The overall prevalence of parasitemia was 33.5% (95% confidence interval [C.I.] 32–34.9); *P. falciparum* was the most prevalent species, either as monoinfection (90.4%; 95% C.I. 88.8–92.1) or combined with *P. malariae* (4.9%; 95% C.I. 3.7–5.9) or *P. ovale* (0.6%; 95% C.I. 0.1–0.9). Only 7.7% (95% CI 6.8–8.6) of households with children under 5 owned an insecticide-treated bednet (ITN), and only 6.8% (95% CI 6.1–7.5) of under-fives slept under an ITN the preceding night. The overall under-5 mortality rate was 147 deaths per 1,000 live births (95% C.I. 141–153) and between clusters was associated with increased *P. falciparum* prevalence; based on the population attributable fraction, 26,488 yearly under-5 deaths were attributable to excess malaria transmission.

**Conclusions:**

Adult *P. falciparum* prevalence is substantial in the DRC and is associated with under-5 mortality. Molecular testing offers a new, generalizable, and efficient approach to characterizing malaria endemicity in underserved countries.

## Introduction

Renewed interest in the eradication of *Plasmodium falciparum* malaria has galvanized efforts to define its spatial epidemiology, which can guide the allocation of resources to areas in greatest need [1], and facilitate evaluation of the effectiveness of control [2]. To this end, several countries have recently generated accurate maps through dedicated Malaria Indicator Surveys [3], [4], and the Malaria Atlas Project (MAP) has meta-analyzed extant parasite prevalence surveys to produce global estimates of *P. falciparum* transmission intensity [5]. Though valuable, such data may be biased both by measurement error and by incomplete global coverage of populations at risk of malaria.

The Democratic Republic of the Congo (DRC) accounts for an estimated 11% of cases of *P. falciparum* malaria in sub-Saharan Africa but there is a paucity of clinical and epidemiologic data [6]. Of high-transmission countries, the MAP data search identified the fewest parasite surveys per land area in the DRC [7], and current data provide little insight into regional variations in transmission intensity [8]. At this time, our understanding of the spatial epidemiology of malaria in the DRC amounts to European cartographers' understanding of the African interior in the late 19^th^ century, when Marlowe, the narrator of Joseph Conrad's *Heart of Darkness*, could remark of what was then the Congo Free State: “At that time there were many blank spaces on the earth … zbut there was one yet – the biggest, the most blank, so to speak – that I had a hankering after [9].”

Herein, we endeavor to populate a blank area of the malaria map by incorporating molecular diagnostics into a nationally-representative household survey in the DRC. By leveraging the infrastructure of the Demographic and Health Survey (DHS), we generate estimates of malaria prevalence, control, and burden in the DRC. Because this approach represents a new, low-cost, and generalizable method of robust malaria surveillance, it offers an opportunity to generate quality malaria epidemiologic data for countries with challenging infrastructure and thus reduce disparities in the quality of malaria surveillance between countries.

## Results

### Survey results and malaria indices

**Figure 1:**
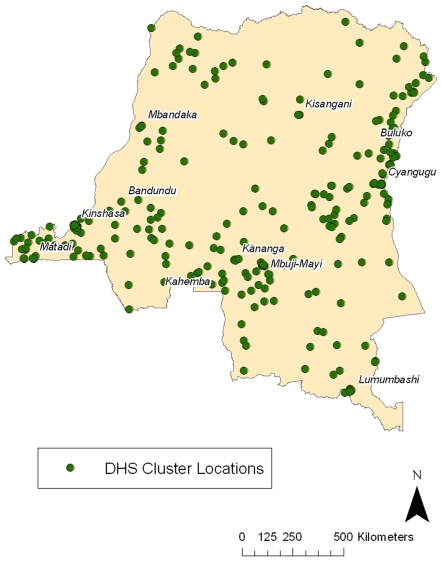
Geographic clusters from which households were selected for sampling.

The 8,886 households from 300 clusters surveyed (Figure 1) yielded data on household-level variables and on 8,992 children born in the previous 5 years resident in those households. Additionally, biometric data were available from 8,838 adult respondents. Survey response rates were 99% for households, 97% for women and 95% for men. Overall, 51.7% of respondents were women and 53.8% lived in a rural community.

**Figure 2:**
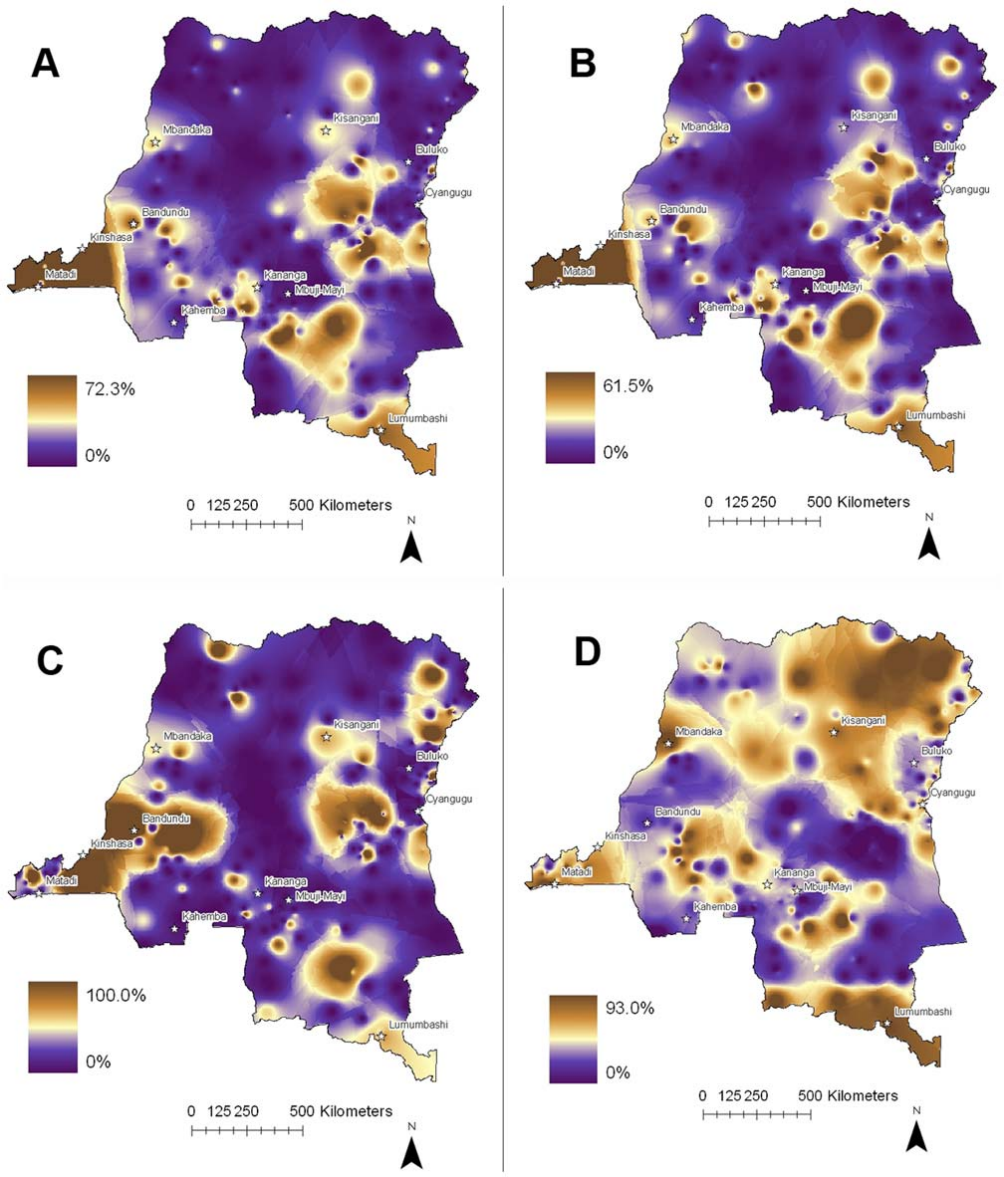
Penetration of malaria control measures. (note difference in visual scales). A. Proportion of households with children under 5 years of age by cluster who reported owning an insecticide-treated net. B. Proportion of children under 5 years of age by cluster that were reported to have slept under an insecticide-treated net the night prior to the survey. C. Proportion of pregnant women by cluster who reported sleeping under an insecticide-treated net the night prior to the survey. D. Proportion of women by cluster who reported taking any antimalarial during their most recent pregnancy within the preceding 5 years.

Malaria control measures targeting children were poorly deployed (Table 1). Of 5,524 households with children less than 5 years of age, only 7.7% possessed an insecticide-treated bednet (ITN; cluster median 5%, interquartile range [IQR] 1–15%), and only 7% of under-fives slept under an ITN the preceding night (cluster median 4%, IQR 0–13%). Clusters with greater ownership and utilization of ITNs for under-fives were notable in the west near Kinshasa and the south near Lubumbashi (Figure 2). Antimalarials were administered to 24% of children under 5 with a fever in the preceding two weeks; therapies containing artemisinin accounted for less than 7%.

**Table 1:** Malaria indices from household survey.

**Households with under-5 children (n = 5524)**	
own no bednet	69.1 (67.4–70.8)
own nontreated bednet	23.2 (21.7–24.8)
own ITN	7.7 (6.8–8.6)
**Under-5 bednet use (n = 7987)**	
did not sleep under bednet previous night	76.6 (75.3–77.9)
slept under untreated bednet	16.6 (15.5–17.8)
slept under ITN	6.8 (6.1–7.5)
**Under-5 clinical illness (n = 7934)**	
recent fever	31 (29.6–32.5)
recent cough	35.5 (34–37.1)
recent diarrhea	16.5 (15.3–17.7)
fever/cough treatment sought (n = 3550)	38.5 (36.2–40.8)
diarrhea treatment sought (n = 1287)	32.6 (29–36.2)
**Under-5 antimalarial treatment for fever (n = 770)**	
Quinine	53.4 (48.1–58.6)
Chloroquine	20.9 (16.9–24.9)
Amodiaquine	16.5 (12.3–20.6)
Fansidar	10.3 (6.3–14.3)
Other antimalarial (including ACTs)	6.4 (4–8.8)
**Pregnancy**	
any antimalarials during last pregnancy (n = 5443)	37.8 (35.9–39.6)
pregnant women who slept under ITN previous night (n = 800)	7.4 (4.7–10.1)
**Parasitemias (n = 8838)**	
any species	33.5 (32–34.9)
*P. falciparum*	32.1 (30.7–33.6)
*P. malariae*	2.9 (2.4–3.5)
*P. ovale*	0.4 (0.2–0.5)

Proportions weighted to account for sampling design. ITN, insecticide-treated bednet. HIV, human immunodeficiency virus. ACT, artemisinin-combination therapy.

Strategies to prevent pregnancy-associated malaria were also rarely implemented (Table 1). Only 7% of pregnant women reported sleeping under an ITN the previous night (cluster median 0; IQR 0–20%), and the geographic heterogeneity in ITN use closely mirrored that of ITN use in under-fives (Figure 2). Under 40% of women who had been pregnant within the previous 5 years reported receiving any antimalarial during their last pregnancy (cluster median 40%, IQR 20–60%).

### Prevalence of parasitemia

Of 8,838 adult respondents, 2,682 (weighted proportion 33.5%; 95% Confidence Interval [C.I.] 32–34.9) were parasitemic (Table 1). Among parasitemias, the most prevalent species was *P. falciparum*, either as monoinfection (90.4%; 95% C.I. 88.8–92.1) or as coinfection with *P. malariae* (4.9%; 95% C.I. 3.7–5.9), *P. ovale* (0.6%; 95% C.I. 0.1–0.9), or all three species (0.1%; 95% C.I. 0–0.3). *P. malariae* was present in 2.9% (95% C.I. 2.4–3.5) of respondents and 8.7% (95% C.I. 7.1–10.3) of parasitemias. *P. ovale* parasitemia was rare overall, as were moninfections with either *P. malariae* (1.2%; 95% C.I. 0.7–1.6) or *P. ovale* (0.1%; 95% C.I. 0.01–0.2).

**Figure 3:**
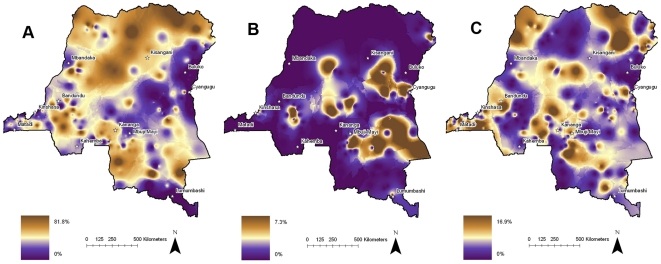
Spatial epidemiology of *P. falciparum*, *ovale*, and *malariae* parasitemias. Parasite prevalence within 300 survey clusters. (note difference in visual scales). A. *P. Falciparum*. B. *P. ovale*. C. *P. malariae.*

There was substantial heterogeneity in parasite rates between clusters (Figure 3). *P. falciparum* prevalences were low in the major cities of Kinshasa, Mbuji-Mayi, Lubumbashi, and Mbadanka, and in the mountainous eastern regions bordering Rwanda and Uganda and the southern region bordering Zambia. Clusters with higher prevalence were marked in rural areas in the interior and in areas bordering Angola in the south and the Central African Republic and the Sudan in the north. *P. falciparum* was present in 284 of 300 clusters, among which the prevalence of *P. falciparum* parasitemia varied from 2.3% to 81.8% (median 31.9%). *P. malariae* was present in 143 clusters distributed across the DRC, and prevalence in these clusters varied from 2% to 16.9% (median 4.3%). *P. ovale* was present in only 28 clusters, largely in the interior and southeast.

### *P. falciparum* endemicity and individual and cluster characteristics

From the lowest to the highest level of *P. falciparum* endemicity (as indicated by the prevalence of *P. falciparum* in adults), the mean cluster altitude decreased and the proportion of rural communities increased substantially (Table 2). Additionally, clusters with higher prevalence tended to have fewer females, be slightly older, and substantially poorer (all p<0.01). Parasitologically, the prevalence of *P. malariae* parasitemias increased with increasing *P. falciparum* prevalence (p<0.01).

**Table 2:** Individual and cluster characteristics by malaria endemicity.

	Cluster *P. falciparum* prevalence in adults				
	0-9.9%(n = 60)	10-24.9%(n = 70)	25-49.9%(n = 104)	≥50%(n = 66)	P-value for trend
**Cluster characteristics**					
Altitude, mean, m	1018 (865–1172)	754 (613–896)	716 (634–798)	658 (541–775)	<0.01
Rural	38.3 (25.9–50.8)	50 (38.1–61.8)	61.5 (52.1–71)	80.3 (70.6–90)	<0.01
**Adults**					
Age, mean, y	29.1 (28.7–29.6)	29.1 (28.7–29.6)	29.9 (29.5–30.3)	30.7 (30.1–31.2)	<0.01
Female sex	54.3 (52.1–56.6)	53.3 (51.2–55.4)	51.1 (49.3–52.8)	48 (45.7–50.4)	<0.01
Wealth index, mean a	3.9 (3.9–4)	3.5 (3.4–3.5)	2.8 (2.8–2.9)	2.3 (2.2–2.3)	<0.01
*P. malariae* prevalence b	1.1 (0.6–1.6)	1.9 (1.4–2.5)	2.9 (2.3–3.5)	3.9 (2.9–4.7)	<0.01
*P. ovale* prevalence b	0.2 (0–0.3)	0.2 (0–0.4)	0.4 (0.1–0.6)	0.6 (0.3–1)	0.08
Women who received any antimalarial during last pregnancy	46.2 (40.4–51.9)	44.2 (37.9–50.3)	36.9 (32.2–41.5)	33 (28.4–37.8)	<0.01
Pregnant women who slept under ITN	18.5 (13–24)	11.4 (6.3–16.6)	13.2 (9.1–17.3)	11.9 (7.1—16.5)	0.22
**Children under 5 years old**					
Household owns ITN	11.4 (8.5–14.4)	10.1 (6.9–13.4)	10.3 (7.1–13.5)	7.9 (4.2–11.5)	0.58
Slept under ITN	9.2 (6.4–12.1)	8.6 (5.7–11.5)	9.6 (6.4–12.7)	7.1 (3.7–10.6)	0.74
Slept under no bednet	66.7 (60–73.1)	73.2 (67.4–79)	73.6 (68.6–78.5)	80.8 (75.8–85.7)	0.01
Recent fever	29.2 (26–32.5)	28.7 (25.8–31.7)	34.6 (31.4–37.8)	34.1 (30.3–38)	0.01
Recent cough	34.5 (31.3–37.6)	32.3 (28.7–35.9)	37.4 (34–40.9)	34.9 (30.4–39.3)	0.23
Recent diarrhea	15.7 (13.8–17.5)	15.5 (13.1–17.9)	16.6 (14.6–18.7)	16.3 (14–18.7)	0.87
Treatment sought for fever/cough	27.4 (23.1–31.7)	25.8 (21.4–30.1)	30.3 (26.5–34.2)	30 (25.2–34.8)	0.4
Treatment sought for diarrhea	37.1 (29.3–45)	41.7 (33.4–50)	35.2 (28.4–42)	28.1 (21–35.2)	0.12
Ever received any vaccination	82.3 (78.1–86.5)	82.7 (77.9–87.5)	78 (73.6–82.4)	76.8 (71.4–82.1)	0.24

Values are expressed as percentages unless otherwise indicated; those in parentheses are 95% confidence intervals. ITN, insecticide-treated bednet.

a Quintiles of 1 (poorest) – 5 (wealthiest) based on household ownership of goods owned and lodging characteristics.

b Includes mixed-species parasitemias.

The penetration of malaria control measures showed mixed associations with endemicity. However, increasing malaria endemicity was associated with increased proportions of fever in children under 5 in the preceding two weeks (p = 0.01). Malaria endemicity was not associated with the frequency of other childhood symptoms, the proportion of children for whom treatment was sought, or the frequency of the receipt of any type of childhood vaccination.

### Malaria endemicity and under-5 mortality

**Figure 4:**
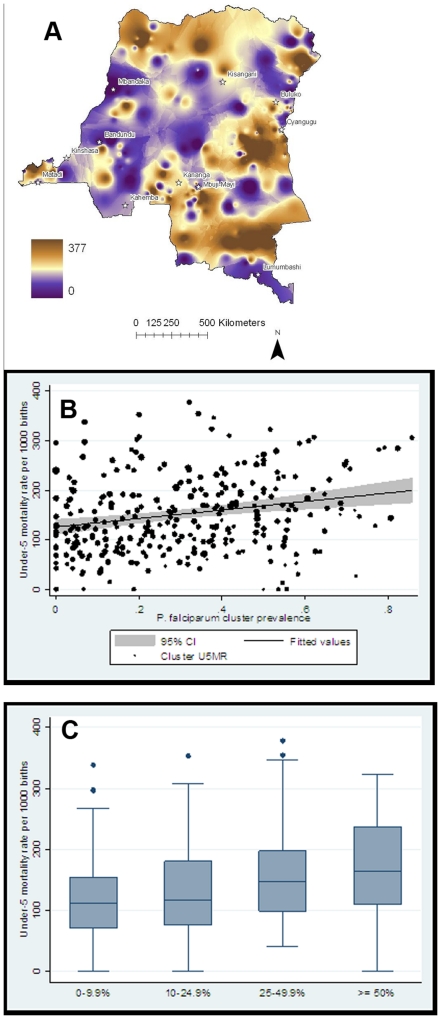
Under-5 mortality and malaria endemicity. A. U5MR (deaths per 1000 live births) by survey cluster. B. Cluster U5MR by adult *P. falciparum* prevalence. Black dots represent actual U5MRs, weighted relative to the number of births in each cluster; black line represents trend line from an unadjusted linear regression model (R^2^ = 0.0538; p<0.001) weighted for differences in numbers of births between clusters (gray area is 95% confidence interval). C. Cluster U5MR by malaria endemicity category. Boxes represent interquartile range, midline is median, individual dots are outliers. p = 0.001 for overall comparison of medians by Kruskal-Wallis analysis of variance.

The overall under-5 mortality rate (U5MR) was 147 deaths per 1,000 live births (95% C.I. 141–153); between clusters, the U5MR ranged from 0 to 378 (standard deviation 77) (Figure 4A). In a linear regression model, increasing cluster *P. falciparum* prevalence was significantly associated with increased U5MR (β = 0.086; R^2^ = 0.0538; 95% C.I. 0.044–0.127) (Figure 4B). Between survey clusters, malaria endemicity was significantly associated with the U5MR, with median (IQR) U5MRs of 111 (72–155), 117 (76–181), 148 (99–198), and 165 (110–237) per 1,000 live births in clusters with parasite prevalences of 0–9.9%, 10–24.9%, 25–49.9%, and ≥50%, respectively (p = 0.001) (Figure 4C). In other pairwise correlations, the cluster U5MR was inversely associated with the frequency of the receipt of any childhood vaccination (p<0.001) and directly associated with the frequency of recent childhood diarrhea (p = 0.006), fever (p<0.001), and cough (p = 0.004).

Among children born in the preceding 5 years, after adjustment for covariates and compared with children born in clusters with parasite prevalences of 0–9.9%, children under 5 died more frequently in clusters with prevalence rates of 10–24.9% (adjusted Odds Ratio [aOR] 1.11; 95% C.I 0.82–1.49), 25–49.9% (aOR 1.38; 95% C.I. 1.05–1.82), and ≥50% (aOR 1.47; 95% C.I. 1.1–1.98) (Table 3). Based on these data and the estimate that 3.28 million births occur in the DRC every year [10], 20.6% of excess under-5 deaths were attributable to malaria in communities with adult parasite prevalence above 10%. With an estimated 553,529 under-5 deaths occurring every year in the DRC [11], we estimate that over 467,000 occur in areas with increased malaria endemicity. Based on our under-5 deaths rates, 128,881 of these deaths exceed what would be observed at the lowest endemicity, and thus intense malaria transmission may be responsible for 26,488 yearly under-5 deaths.

**Table 3:** Results of a multilevel model of cluster-level indicators on under-5 deaths.

	Adjusted Odds Ratio (95% C.I.)	p-value
***P. falciparum*** ** prevalence** a		
0.-9.9%	REF	—
10-24.9%	1.11 (0.82–1.49)	0.501
25-49.9%	1.38 (1.05–1.82)	0.021
> = 50%	1.47 (1.1–1.98)	0.01
**Prevalence of vaccination receipt** b		
1^st^ quartile	REF	—
2^nd^ quartile	0.76 (0.59–0.99)	0.04
3^rd^ quartile	0.86 (0.67–1.1)	0.235
4^th^ quartile	0.47 (0.36–0.63)	<0.001
**Diarrhea prevalence** c		
1^st^ quartile	REF	—
2^nd^ quartile	1.03 (0.78–1.36)	0.836
3^rd^ quartile	1.16 (0.88–1.54)	0.287
4^th^ quartile	1.02 (0.76–1.37)	0.894
**Fever prevalence** c		
1^st^ quartile	REF	—
2^nd^ quartile	1.02 (0.77–1.35)	0.902
3^rd^ quartile	1.20 (0.88–1.64)	0.246
4^th^ quartile	1.13 (0.80–1.59)	0.502
**Cough prevalence** c		
1^st^ quartile	REF	—
2^nd^ quartile	0.88 (0.65–1.18)	0.38
3^rd^ quartile	0.97 (0.72–1.31)	0.865
4^th^ quartile	1.09 (0.78–1.51)	0.616

C.I.: confidence interval. Odds ratios are adjusted for other covariates. 1^st^ quartile are clusters with the lowest proportion of the indicated variable, 4^th^ quartile with the highest proportion of the indicated variable. All children born to female respondents in or since 2003 were included (n = 8290).

a Proportion of adults in each cluster who were parasitemic with *P. falciparum* by real-time PCR testing.

b Proportion children under 5 in each cluster who had ever received any vaccination for any disease (as reported by the child's mother).

c Proportion of children under 5 in each cluster who had diarrhea, fever, or cough in the preceding two weeks, (as reported by the child's mother).

## Discussion

High throughput PCR analyses of leftover dried blood spots enabled us to map and quantify malaria endemicity in the DRC without the need for a dedicated and complex malaria indicator survey. Overall, the point prevalence of malaria parasitemia was over 33% in adults, and parasite prevalence within survey clusters was associated with traditional determinants of malaria endemicity and with increased under-5 mortality. These associations confirm the utility of parasite prevalence as a measure of malaria endemicity.

To our knowledge, this is the first nationally-representative malaria survey in sub-Saharan Africa to employ molecular diagnostics, and the first attempt to describe the epidemiology of malaria in this geographic area since 1951 [12]. These data add substantially to current attempts to describe malaria endemicity, most notably the Malaria Atlas Project (MAP) [5]. Unfortunately, the MAP produced high levels of uncertainty for prevalence estimates in the DRC owing to a paucity of data for central Africa [5], relied on studies that employed microscopy for parasite detection which routinely underestimates the true parasite rate [13], and utilized parasite rate surveys which were derived from data sources up to over 20 years old. Moreover, many highly malarious countries often lack both any reliable malaria data and the infrastructure to undertake complex surveillance. Integrating molecular surveillance into DHS surveys, which have been conducted in 90 countries, offers an opportunity to expand quality malaria surveillance to underserved countries.

Our method of parasite detection and mapping offers substantial advantages compared with existing methods. Molecular assays for malaria are substantially more sensitive than detection using either blood smears or rapid diagnostic tests [13] and offer the ability to more definitively detect mixed-species infections with non-*falciparum* species [14]. Furthermore, our approach generates data on the geographic limits of malaria transmission in a fashion that is both methodologically reproducible and can reflect changes in malaria epidemiology over time if incorporated into interval surveys. Parasite rates have been proposed as benchmarks for renewed malaria control efforts [15]. Malaria indicator surveys can provide such data [3], [4], but are resource-intensive and unfeasible for many malaria endemic countries. The incorporation of molecular testing into the DHS exploits the size, sophistication, and frequency of an existing effort, and can provide malaria data in less-developed countries where the malaria burden falls most heavily.

Successful malaria control has been highly dependent on preventive measures and effective therapy [16], [17], [18], but the penetration of these initiatives in the DRC was poor: only 8% of households with children less than 5 years of age owned insecticide-treated bednets (ITNs), and less than 7% of children under 5 slept under an ITN. Interventions to prevent pregnancy-associated malaria – a major contributor to low-birth weight and maternal and infant mortality – were similarly limited, with over 60% of women denying the receipt of any antimalarial during their last pregnancy and only 7% of currently pregnant women reporting having slept under an ITN the previous night. These numbers contrast with official policies that have called for the free distribution of ITNs to children and pregnant women since 2006 and the use of intermittent preventive therapy for malaria during pregnancy since 2002 [6]. Futhermore, fewer than 7% of children treated with an antimalarial for fever were administered therapy containing artemisinin, despite the adoption in 2005 of artesunate-amodiaquine as first-line recommended therapy [6]; additionally, there was substantial use of poorly-tolerated (quinine) [19] and ineffective (sulfadoxine-pyrimethamine) antimalarials [20]. Geographic parsing of the rates of uptake of recommended control measures between survey clusters demonstrated substantial heterogeneity, with penetration generally less successful in less developed and accessible regions. Though sobering, these numbers indicate substantial opportunities to improve malaria control.

The adult parasite rates of 34% overall and 82% in some clusters concur with previous studies of PCR-detectable parasitemia in other highly-endemic sub-Saharan African settings [21], [22], [23], [24], [25], [26], [27], [28]. The clinical relevance of these largely asymptomatic parasitemias in adults is indicated by the association within survey clusters between *P. falciparum* prevalence and under-5 mortality (U5MR). Reducing U5MR is a global public health priority but progress in the DRC is lacking [29]. At present, more under-5 children die every year in the DRC than any other country except Nigeria and India, with deaths principally attributed to pneumonia (20%), diarrhea (19%), and malaria (17%) [11]. We estimate that over 27,000 yearly under-5 deaths in areas with elevated endemicity are attributable to malaria. These deaths underestimate the total burden of malaria on under-fives in the DRC, but they suggest that reductions in malaria endemicity will be accompanied by substantial reductions in child deaths.

Though unmeasured factors may account for these differences in U5MR between levels of malaria endemicity, we incorporated covariates that address other major contributors to childhood mortality – lower respiratory tract infection and diarrhea; treatment-seeking behavior for fever, cough, and diarrhea; and the receipt of any vaccination – in order to capture a reasonable degree of variation in clinical syndromes, health system access, and public health infrastructure that influence childhood mortality. Additionally, the association between malaria endemicity and U5MR may be complicated by the complexity of the relationship between endemicity and the clinical consequences of malaria infection [30], [31], [32]. However, across a range of transmission levels malaria-specific mortality concentrates in the under-5 population [33].

This report is subject to several additional limitations. Comparing our results with other prevalence studies is complex because we measured parasite rates using PCR testing in adults, while other studies typically measure microscopic parasitemias in children [34]. However, these parasite rates are associated with common determinants of malaria transmission (altitude, wealth, urban and rural residence) and with under-5 mortality, demonstrating both their utility as an index of endemicity and their public health significance. Additionally, our estimation of U5MR differs from others that employ a cohort life table approach. This approach was not feasible to calculate U5MRs on a relatively small cluster level, and thus we employed a recently validated, simplified method of U5MR estimation [35]. We did not explore the effect of HIV infection on childhood mortality or other indices, though the prevalence of HIV in adults surveyed in the 2007 DRC DHS was only 1.3% [36]. Finally, we cannot fully account for seasonal variation in malaria prevalence, though recent rainfall was not associated with parasitemia in a multivariate model (Messina J, manuscript submitted).

The integration of molecular malaria testing Demographic and Health Surveys offers a cost-effective opportunity to generate robust data in order to guide the allocation of resources and measure control initiative effectiveness. Such initiatives are succeeding in multiple sub-Saharan African settings, including Kenya [37], the Gambia [16], and Tanzania [17]. Comparatively less interest and enthusiasm for control efforts have been directed at highly-endemic countries. This work in the DRC populates a blank space on the malaria map to inform future efforts to control malaria and reduce mortality in the most intensely malarious regions on earth.

## Materials and Methods

### Ethics statement

All survey respondents provided verbal informed consent for the collection of blood spots in one of the five main languages spoken in the DRC per DHS protocol. Consent was verbally acquired owing to both limited literacy levels in rural provinces as well as the need for immediate de-identification of all data at the conclusion of the interview to ensure the confidentiality of the publicly-accessible survey datasets. Consent procedures, survey administration, and blood sample collection were approved by the Ethics Committees of Macro International and the School of Public Health of the University of Kinshasa, and testing for malaria parasites was approved by the Institutional Review Board of the University of North Carolina.

### Survey methodology

The 2007 DHS was designed to provide nationally-representative data on population, health, and social indices for program planning and impact evaluation. It was the first such survey conducted in the DRC and represented a collaboration between the DRC Ministries of Health and Planning and Macro International, with funding provided by the United States' Agency for International Development United Nations, the World Bank, and the United Kingdom's Department for International Development.

The DHS employed a stratified two-stage cluster design based on DRC census data obtained before the national election in 2006. The survey's sampling units were 300 randomly selected geographic clusters (Figure 1), from which 9000 households were selected for inclusion; all women aged 15 to 49 years within these households were surveyed, and, in half of the households, men aged 15 to 59 were surveyed. All men and half of the women and men were consented for collection of blood spots. Three questionnaires, translated into the four main languages spoken in the DRC, were employed: for the household, for women, and for men. The household survey mainly served to identify men and women for possible individual inclusion, and included basic household data. Topics in the female questionnaire included birth history, childhood health indices and vital status, and a malaria module; the male questionnaire was substantially reduced. The DHS was conducted in February and March for clusters in and near Kinshasa, and between May and August for the balance of the DRC.

Blood spots were collected from adult women and men in half of the surveyed households, primarily for estimating the seroprevalence of HIV. After consent was obtained, blood was collected in multiple spots on a single filter paper from each participant and placed in a drying box with desiccant overnight. After transfer to sealed plastic bags with desiccant and humidity indicators, they were placed into coolers prior to transport to Kinshasa. From each card, three 0.3 inch discs were punched and deposited in a single well of a plastic 96-well sample plate, which, when full, was sealed with a plastic cover, placed in a sealed plastic bag with desiccant, and stored at room temperature.

### Molecular assays

Plates of punched blood spots were transferred from Kinshasa to the University of North Carolina, where genomic DNA (gDNA) was extracted using the invitrogen PureLink 96 Kit (invitrogen, Carlsbad, CA) using a vacuum manifold. In the final step, gDNA was eluted using 200 uL of elution buffer into a fresh 96-well sample plate, and sample plates were sealed securely and stored at -20C prior to further processing.

Molecular testing employed two real-time PCR assays that target the 18S ribosomal DNA sequence of Plasmodia. Samples were initially amplified in a real-time PCR assay that detects all species of Plasmodia [38], which included 4uL of gDNA in a 25 uL reaction volume. Samples demonstrating amplification in this pan-species assay were then amplified in a speciation assay that targets species-specific sequences of *P. falciparum*, *P. ovale*, and *P. malariae*, as well as that of a human control gene [38]; this assay consisted of two duoplex assays run in parallel, each of 25 uL with 2 uL of gDNA Threshold lines were set manually based on controls to exclude background fluorescence upon inspection of the delta Rn plots over 40 cycles; to optimize sensitivity of the assays, any amplification of target was interpreted as positive.

All reaction plates included negative controls (with molecular-grade water in place of template) and positive controls: *P. falciparum* gDNA for the pan-species assay and mixtures of *P. falciparum*, *P. ovale*, *P. malariae*, or human DNA for the speciation assay, as appropriate. The first 867 samples were tested in duplicate; because of the lack of discordant results, the balance of samples was tested singly in each assay. For quality control, a random set of 5% of all gDNA samples was retested in the pan-species assay, and discordant repeat pan-species results were resolved by repeating the speciation assay. Ultimately, 8 specimens (of 520 re-tested) that were initially pan-species negative were determined to harbour *P. falciparum* after quality control testing. All reactions were performed on an ABI 7300 System (Applied Biosystems, Foster City, CA) and analyzed using the ABI 7300 Sequence Detection Software (v1.3). Reaction plates were prepared using an epMotion 5070 system (eppendorf, Hamburg, Germany). To minimize the risk of sample contamination, filtered pipet tips were exclusively employed in all steps, and separate work areas were maintained for punching discs from blood spots, extracting gDNA, preparing reaction mixtures, and assembling reaction plates.

In subsequent analyses, malaria parasitemia positivity was defined as the presence of any species of *Plasmodium* by both the pan-species and the speciation real-time PCR assays.

### Analyses of survey and malaria data

Population-level proportions of the penetration of malaria control measures (bednet use among children and pregnant women, effective antimalarial use, and receipt of antimalarials during pregnancy), the prevalence of childhood illnesses, and the prevalence of parasitemias were calculated using sampling weights to account for survey design. For children born in the preceding 5 years, women were queried whether the child slept under a bednet the previous night; ever received any vaccination; experienced fever, cough or diarrhea in the preceding 2 weeks; was administered treatment for fever, cough, or diarrhea; and was administered an antimalarial for fever. Additionally, heads of households were queried about the residence of children under 5 and the possession and type of bednet, and women were queried about pregnancy status and their personal bednet use.

To analyze geographic variation of the adoption of malaria control measures in children and pregnant women, cluster-level indicators were generated. Within each cluster, we calculated the prevalence of parasitemia with each species and, using survey data, the cluster-level frequencies of adherence to malaria control measures among children and pregnant women. Using ArcGIS (v9.3, ESRI, Redlands, CA, USA), individual maps of parasite rates and rates of uptake of control measures were generated using inverse-distance weighting to smooth surfaces across unmeasured locations between survey clusters. Additionally, we tested the cluster parasite prevalences by species for spatial autocorrelation by examining the significance of global Moran's I values based upon row-standardized inverse distance weights matrices. Prior to analysis, cluster coordinate locations were randomly offset by 2 kilometers (in urban clusters) or 5 kilometers (in rural clusters) to maintain privacy.

To examine associations between malaria endemicity and cluster-level indicators, the proportion of adults in each cluster that were parasitemic with *P. falciparum* served as an index of malaria endemicity. Clusters were grouped into categories of *P. falciparum* prevalence that approximated quartiles: 0–9.9%, 10–24.9%, 25–49.9%, and ≥50%. Bivariate associations between malaria endemicity (the main independent variable) and cluster-level demographics, adoption of malaria control measures, and childhood illnesses were assessed with one-way analysis of variance or the chi-squared test for continuous or categorical outcome variables, respectively.

### Malaria endemicity and childhood mortality

Surveyed women were queried about their total number of births and about the vital status of all births. From complete birth histories, the under-5 mortality rate (U5MR) was calculated on national and cluster levels as the fraction of children ever born to surveyed women that died prior to 60 months of age. Cluster-level U5MR was mapped as above. This approach is biased towards underestimating U5MR because it does not account for the foreshortened time of exposure among children less than 5 years of age at the time of survey. Nevertheless, due to the small sample size at the cluster level, it was deemed a more appropriate method than the life table technique, and it has been validated as an accurate estimate compared with more traditional methods [35].

To assess potential confounders of the main effect of malaria endemicity and U5MR, cluster-level proportions were generated for prevalence of the receipt of any vaccination (among all live births); the prevalences of fever, cough, and diarrhea (among all living children); and the prevalence of medical treatment sought for fever, cough, or diarrhea (among children with respective symptoms).

To evaluate the overall influence of malaria endemicity (as reflected by the cluster *P. falciparum* prevalence) on cluster U5MR, linear regression was employed; the independent variable was the cluster parasite prevalence as a continuous measure and the dependent variable was the cluster U5MR. Differences in median U5MR by cluster malaria endemicity (categorized as above) were compared by the Kruskal-Wallis analysis of variance due to the non-parametric distribution of cluster U5MRs.

To estimate the population attributable fraction (AF_p_) of parasite prevalence on under-5 mortality, adjusted odds ratios (aORs) for under-5 death were calculated using logistic regression. The model incoporated child health covariates that were significantly associated with U5MR in bivariate testing. Because the data were clustered and the model included cluster-level independent variables and an individual-level dependent variable, a multilevel random-intercept logistic regression model was employed to account for the potential interdependence of observations within cluster groups (xtlogit in Stata/IC version 10.0, StataCorp, College Station, TX) [39]. In this model, the main independent variable was the categorized adult parasite prevalence within each cluster; the dependent variable was the death of a child born in the preceding 5 years. As covariates, the model included cluster-level indicators of the proportion of children under 5 who had ever received any vaccination and who had suffered diarrhea, cough, or fever in the preceding two weeks. Clusters with missing values were excluded from the model. The AF_p_ for under-5 deaths by each parasite prevalence category was then estimated using the aORs and the fraction of births within each parasite prevalence category [40]. This fraction of births across the DRC as a whole was extrapolated from the distribution of births within surveyed clusters, assuming that the survey was nationally-representative.

### Statistical analyses and data management

All reported p-values are two-sided; a p-value of less than 0.05 was considered appropriate to reject the null hypothesis for each analysis. Molecular data were imported directly into a FileMaker Pro database (version 10, FileMaker, Inc, Santa Clara, CA) and later merged with survey data in Stata/IC; all statistical analyses were subsequently performed with Stata/IC.
